# Identification of Human Motion Using Radar Sensor in an Indoor Environment

**DOI:** 10.3390/s21072305

**Published:** 2021-03-25

**Authors:** Sung-wook Kang, Min-ho Jang, Seongwook Lee

**Affiliations:** School of Electronics and Information Engineering, College of Engineering, Korea Aerospace University, Goyang-si 10540, Gyeonggi-do, Korea; sys77750@kau.kr (S.-w.K.); jmh17360@kau.kr (M.-h.J.)

**Keywords:** motion identification, radar sensor, spectrogram, target classification

## Abstract

In this paper, we propose a method of identifying human motions, such as standing, walking, running, and crawling, using a millimeter wave radar sensor. In our method, two signal processing is performed in parallel to identify the human motions. First, the moment at which a person’s motion changes is determined based on the statistical characteristics of the radar signal. Second, a deep learning-based classification algorithm is applied to determine what actions a person is taking. In each of the two signal processing, radar spectrograms containing the characteristics of the distance change over time are used as input. Finally, we evaluate the performance of the proposed method with radar sensor data acquired in an indoor environment. The proposed method can find the moment when the motion changes with an error rate of 3%, and also can classify the action that a person is taking with more than 95% accuracy.

## 1. Introduction

Recently, as the center frequency used by radar sensors increases, the size of the radar is getting smaller and its range resolution is getting higher. Keeping pace with these changes, radar sensors have been used for various purposes. The radar sensors are mounted on vehicles to provide safety and convenience to drivers [1], and are also embedded on smart phones to recognize human faces [2,3]. In addition, they are used to locate people inside a vehicle [4,5] and to estimate biometric data, such as respiration rate and heart rate [6,7]. As such, radar sensors are gradually expanding their applications to fields that are closely related to our lives.

Among them, the demand for the use of radar in indoor environments is increasing. Until now, vision sensors and red, green, and blue (RGB) images have been widely used to recognize human motion and monitor people’s movements [8]. However, these camera-based human recognition methods are vulnerable in terms of privacy protection in that they acquire images of each individual. On the other hand, because the radar sensor obtains the shape of an object through the reflected radio waves, it has the advantage of being relatively free from the aforementioned problems. Recently, various studies have been conducted to detect people indoors using radar sensors. For example, a microwave Doppler radar sensor was used to detect people moving behind the wall [9], and an impulse radio ultra-wideband (UWB) radar was also used to identify the location of a person in a room [10].

Going further from studies that identify people’s location, research on classifying human motions indoors with the radar systems has been actively conducted. In [11], the authors recognized human motions by applying the variational mode decomposition to the received UWB radar signal. In addition, various human motions were classified according to micro-Doppler characteristics under micro-motion interference in [12]. Moreover, studies on classifying human motions using deep learning models trained with radar data sets have been actively conducted [13]. For example, a method of classifying arm motions by applying deep learning techniques has also been proposed [14]. Based on these studies, we propose a more advanced method that finds the moment when a person’s motion changes and at the same time discriminates the motion using a radar sensor in an indoor environment.

In our work, we use a frequency-modulated continuous wave (FMCW) radar sensor with a center frequency of 61 GHz and a bandwidth of 6 GHz, respectively. This FMCW radar has the advantage of having a high range resolution while consuming low power [15]. In addition, unlike studies using Doppler radars [3,12,14], the characteristics of the distance change over time of an object can be recognized by the FMCW radar system. Then, we install the FMCW radar sensor and acquire sensor data on various human motions, such as standing still, walking, running, and crawling. In our measurement, we also accumulate radar sensor data for a person performing two motions in succession.

To identify the motion of a person, it is necessary to understand the change in radar signal characteristics over time. Therefore, we generate a spectrogram from the received radar signal and suppress the direct current (DC) component and the static clutter to extract only motion information from it. The extracted spectrogram is not used as it is, but the cropped spectrogram is used considering the area where the human exists. Then, two major signal processes are performed in parallel in our proposed method. First, statistical characteristics appearing in the cropped spectrogram are used to find out the moment when the movement changes. Second, to distinguish each motion, a convolutional neural network (CNN)-based classifier is trained with the cropped spectrogram. Recently, many studies have been conducted to classify targets by training radar data with the CNN. For example, CNN models to classify hand gestures were designed [16,17], and CNN-based classifiers for classifying radar waveforms were also proposed [18,19]. In our study, different CNN structures are determined according to the number of classes to be distinguished, and then classification performance is evaluated for each structure.

The contributions of our research can be summarized as follows. Most studies focus on classifying single motions based on the spectrograms [20,21,22]. However, our proposed method also can identify two consecutive motions because we also use the sensor data of the part where the motion changes. In [23], even though the CNN was trained with data on changing motion, its accuracy was reported to be very low. In addition, because we use the change in the statistical characteristics of the radar signal over time, we can determine the moment when the motion changes, which is not considered in most related studies.

The remainder of this paper is organized as follows. In Section 2, the millimeter wave band FMCW radar sensor and signal measurement environment are introduced. In addition, preprocessing for suppressing the DC component and static clutter from the accumulated radar signal are described. Next, a method of determining the moment when a person’s motion changes and discriminating the motion is presented in Section 3. The performance of the proposed method is also evaluated in Section 3. Finally, we conclude this paper in Section 4.

## 2. Radar Sensor Data Acquisition and Signal Preprocessing

### 2.1. FMCW Radar Sensor

**Figure 1:**
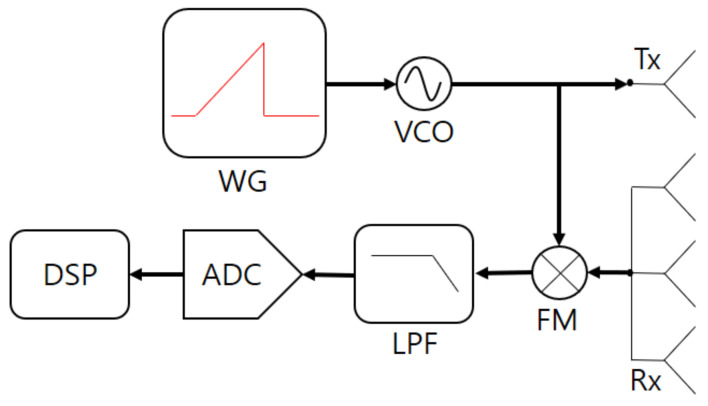
Configuration of the frequency-modulated continuous wave (FMCW) radar system.

**Figure 2:**
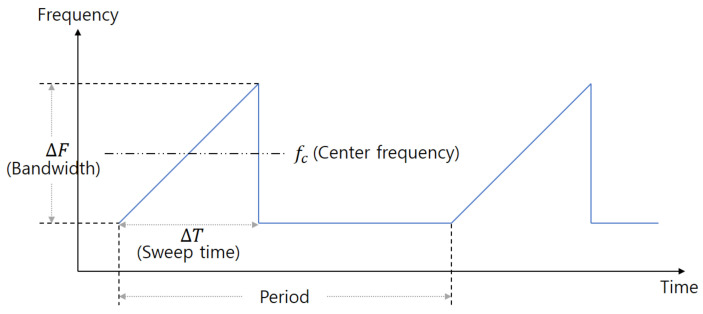
FMCW radar signal generated from the WG.

The configuration of the FMCW radar system we used in the experiment is shown in Figure 1. This system consists of a transmit antenna (Tx), receiving antennas (Rxs), a waveform generator (WG), a voltage-controlled oscillator (VCO), a frequency mixer (FM), a low-pass filter (LPF), an analog-to-digital converter (ADC), and a digital signal processor (DSP). The WG generates a baseband FMCW radar signal whose frequency changes over time as shown in Figure 2. Here, one waveform in one period is also called a chirp signal.

In this system, the baseband signal passed through the ADC [24] can be expressed as

(1) $$x\left[n\right]=\sum_{l=1}^{L}\alpha_{l}\exp\left[j2\pi \left\{\left(\frac{2\Delta F}{c\Delta T}d_{l}+\frac{2f_{c}}{c}v_{l}\right)nT_{s}+\frac{2f_{c}}{c}d_{l}\right\}\right],$$

where l(l=1,2,…,L) is the index of the object and $\alpha_{l}$ indicates the amplitude of the baseband signal. In addition, $d_{l}$ and $v_{l}$ denote the relative distance to the *l*th object and the relative velocity of the *l*th object, respectively. In addition, ΔF and ΔT represent the bandwidth and the sweep time of each chirp, which is illustrated in Figure 2. Finally, in the ADC, one baseband chirp signal is sampled *N* times at a sampling interval of $T_{s}$.

When applying the Fourier transform [25] to the signal of Equation (1), we can obtain a baseband signal in the frequency domain, which can be expressed as

(2) $$X\left[k\right]=\sum_{n=0}^{N-1}x\left[n\right]\exp\left(-j2\pi \frac{n}{N}k\right),$$

where k(k=0,1,…,K−1) is the frequency index. By accumulating the frequency-domain baseband signal over time, we can obtain the spectrogram, which shows the change in distance of an object over time [26]. In other words, in the FMCW radar system, the detection result in the frequency domain can be interpreted as that in the distance domain [24]. The spectrogram of the baseband signal accumulated over $N_{p}$ periods can be expressed as

(3) $$X^{\left(N_{p}\right)}=\left[X^{\left(1\right)},\;X^{\left(2\right)},\;\ldots ,\;X^{\left(N_{p}\right)}\right],$$

where $X^{\left(i\right)}={\left[X^{\left(i\right)}\left[0\right],\;X^{\left(i\right)}\left[1\right],\;\ldots ,\;X^{\left(i\right)}\left[K-1\right]\right]}^{T}$ is the frequency-domain baseband signal in the form of a vector and *i* indicates the index of the period. For example, because one period of our radar signal is 50 ms, $N_{p}$ becomes 20 in the spectrogram accumulated over 1 s.

### 2.2. Measurement Environment

**Figure 3:**
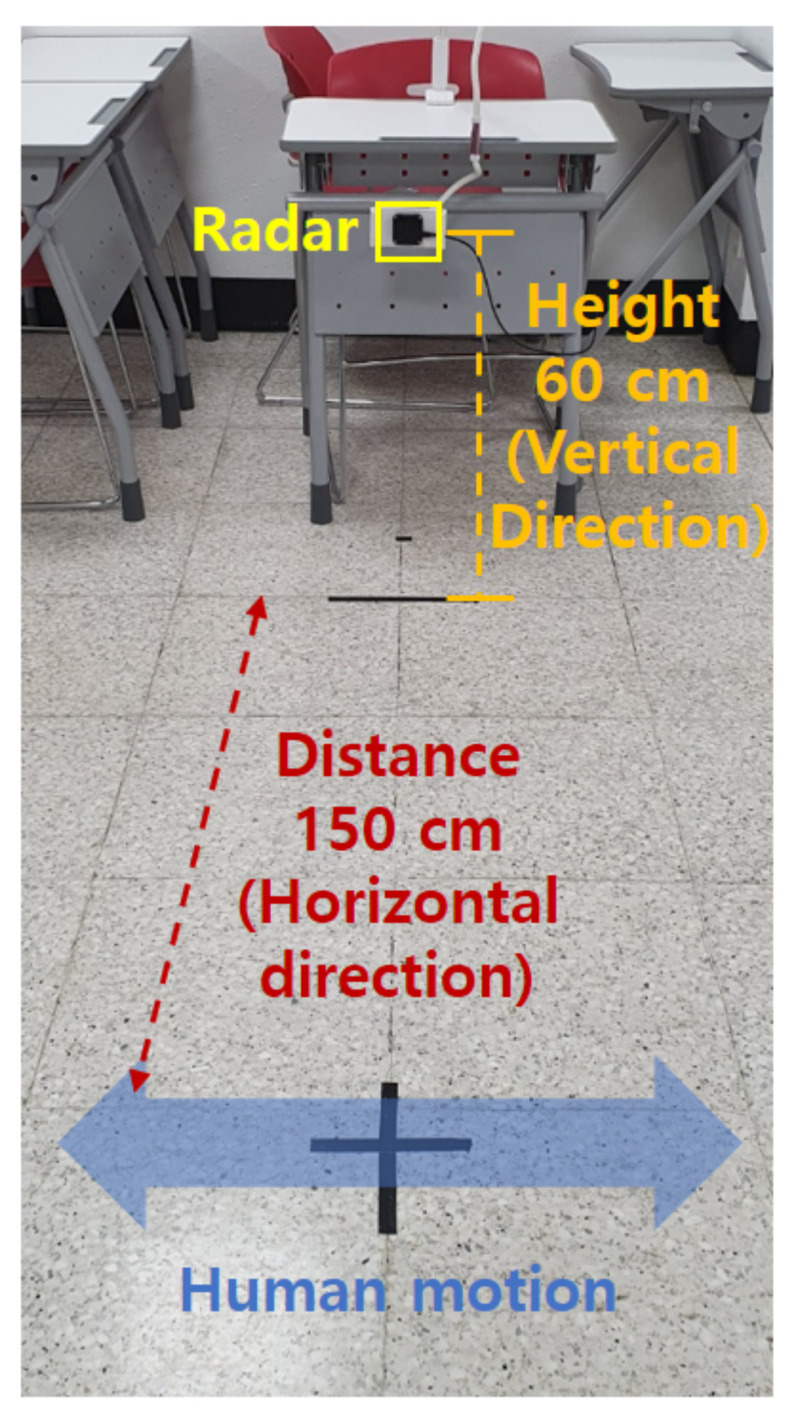
Signal measurement in an indoor environment.

**Figure 4:**
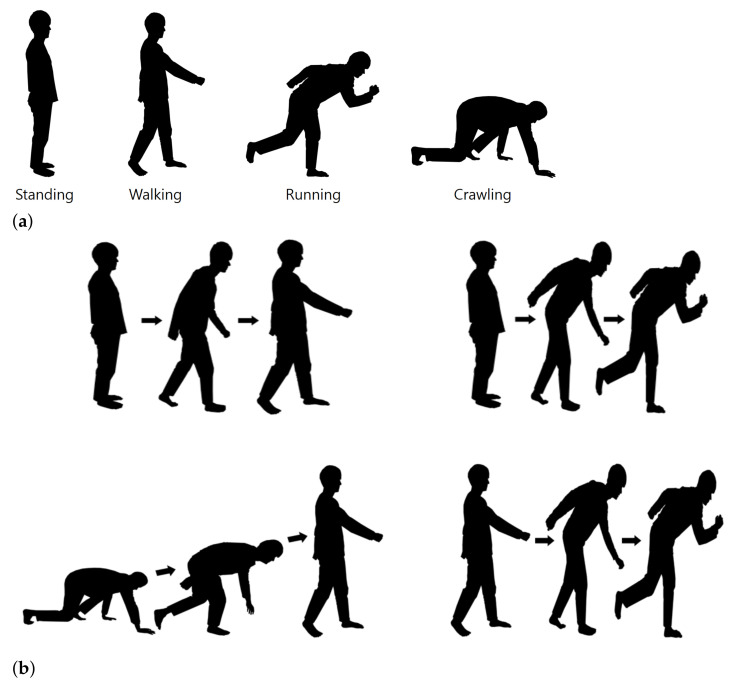
Examples of motions: (**a**) Performing only one motion. (**b**) Performing two motions in a row.

We conducted experiments in an indoor environment as shown in Figure 3, using the radar sensor described in Section 2.1. First, to determine the proper installation height of the radar, the radar height was increased by 20 cm from the ground (e.g., 0 cm, 20 cm, 40 cm, *…*). Then, in consideration of the intensity of the received signal and the effect of clutter caused by the ground, the radar installation height was set to 60 cm. For the fixed height, we have accumulated radar sensor data for four different motions (e.g., standing still, walking, running, and crawling) of several different people. In the first measurement, the radar data was acquired while each person maintained one motion, and in the second measurement, data was acquired while performing two motions in succession, as shown in Figure 4. We accumulated over 500 spectrograms for each single motion and two consecutive motions.

### 2.3. Preprocessing of Radar Sensor Data

The spectrogram described in Section 2.1 contains the DC component of the baseband signal and the static clutter. To extract only the signals corresponding to human motions, such unnecessary signals must be removed. In other words, a signal preprocessing step to remove those unnecessary signals is required before generating the input data used for motion identification. Thus, we propose a method to remove the DC component and the static clutter that degrade human motion detection performance. Various methods, such as a mean subtraction method [27], a range profile subtraction method [27], a linear trend subtraction method [28], can be applied to suppress the DC component and the radar clutter. Among these methods, we applied the mean subtraction method, which has low computational amount and excellent clutter suppression performance. The process of applying the mean subtraction method to the spectrogram can be expressed in two consecutive steps. The DC component suppression can be expressed as

(4) $$Y^{\left(N_{p}\right)}=X^{\left(N_{p}\right)}-J_{K,\;1}\times \frac{1}{K}\left[\sum_{k=0}^{K-1}X^{\left(1\right)}\left[k\right],\;\sum_{k=0}^{K-1}X^{\left(2\right)}\left[k\right],\;\ldots ,\;\sum_{k=0}^{K-1}X^{\left(N_{p}\right)}\left[k\right]\right],$$

and then the static clutter suppression can be expressed as

(5) $$\begin{array}{ccc} Z^{\left(N_{p}\right)} & = & Y^{\left(N_{p}\right)}-\frac{1}{N_{p}}{\left[\sum_{i=1}^{N_{p}}X^{\left(i\right)}\left[0\right],\;\sum_{i=1}^{N_{p}}X^{\left(i\right)}\left[1\right],\;\ldots ,\;\sum_{i=1}^{N_{p}}X^{\left(i\right)}\left[K-1\right]\right]}^{T}\times J_{1,\;N_{p}}, \end{array}$$

where $J_{P,\;Q}$ represents a matrix in which all elements are 1 and its size is P×Q.

**Figure 5:**
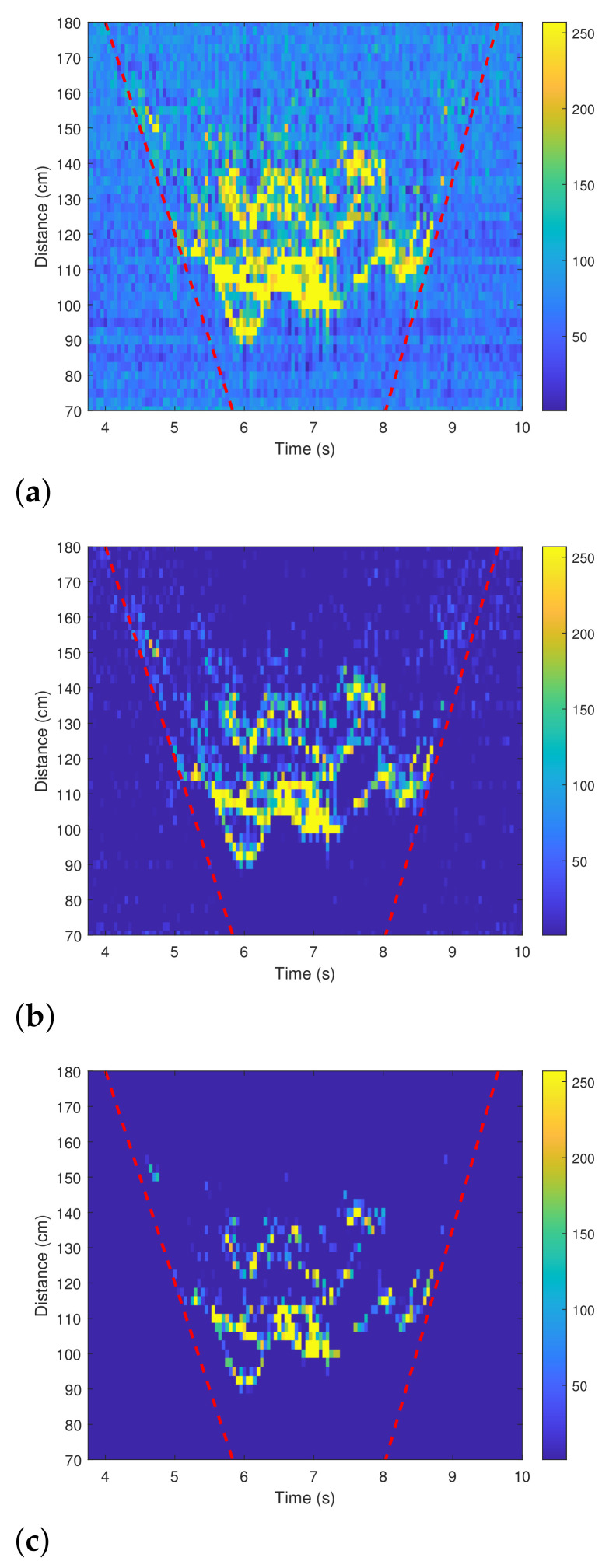
Spectrograms: (**a**) Before applying the mean subtraction method ($X^{\left(N_{p}\right)}$). (**b**) After removing the DC component ($Y^{\left(N_{p}\right)}$). (**c**) After removing the DC component and the static clutter ($Z^{\left(N_{p}\right)}$).

For example, Figure 5a shows the raw spectrogram when a person is crawling in the field of view (FOV) of the radar. The FOV is also indicated by red dotted lines in the figure. Then, Figure 5b shows the spectrogram after the DC component has been removed. In addition, the spectrogram with the clutter also removed is shown in Figure 5c. When compared with Figure 5a, only the strength of the signal corresponding to the human motion remains strong in Figure 5c.

## 3. Proposed Human Motion Identification Method

### 3.1. Generating Input Data

**Figure 6:**
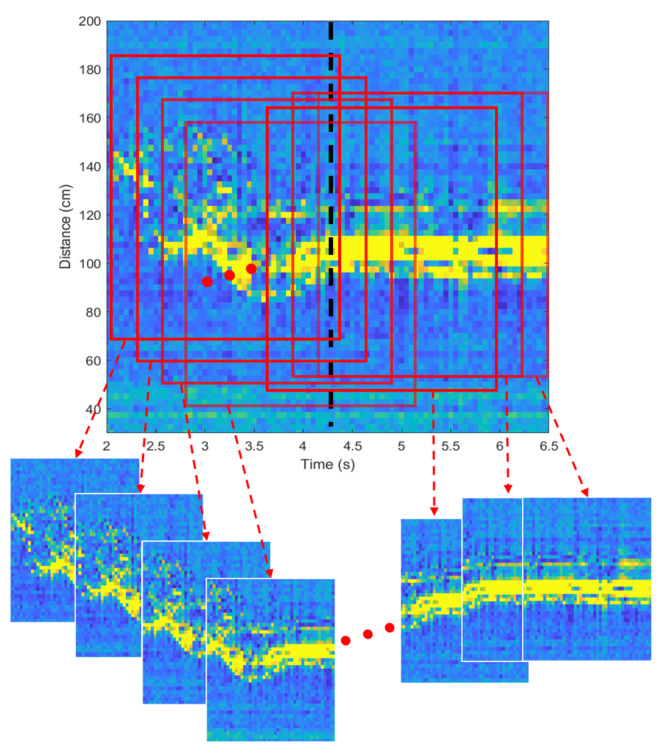
Generating cropped spectrograms.

As mentioned earlier, our proposed method is composed of two parallel signal processing: one is to find the moment when the motion changes, and the other is to discriminate the type of motion. In both signal processing, the identical spectrogram is used as input. However, $Z^{\left(N_{p}\right)}$ is not used as it is, but the cropped spectrogram (i.e., $Z_{c}^{\left(N_{p}\right)}=\left[Z_{c}^{\left(1\right)},\;Z_{c}^{\left(2\right)},\;\ldots ,\;Z_{c}^{\left(N_{p}\right)}\right]$) is used considering the area where the human exists. In other words, the size of the spectrogram is reduced in consideration of the elapsed time and the distance spread of the object. In the data set we accumulated, the distance range of human motions was spread up to 100 cm. Therefore, we get 40 values on the distance axis of the spectrogram because the range resolution of our FMCW radar system is 2.5 cm. On the time axis, the spectrogram for 1.5 s was cropped by applying a rectangular window. As a result that one period of our radar signal is 50 ms, 30 values were obtained. Finally, this matrix data of size 40 × 30 is extracted every time slot (i.e., 50 ms), and the process of generating the cropped input data from the entire spectrogram is illustrated in Figure 6.

**Figure 7:**
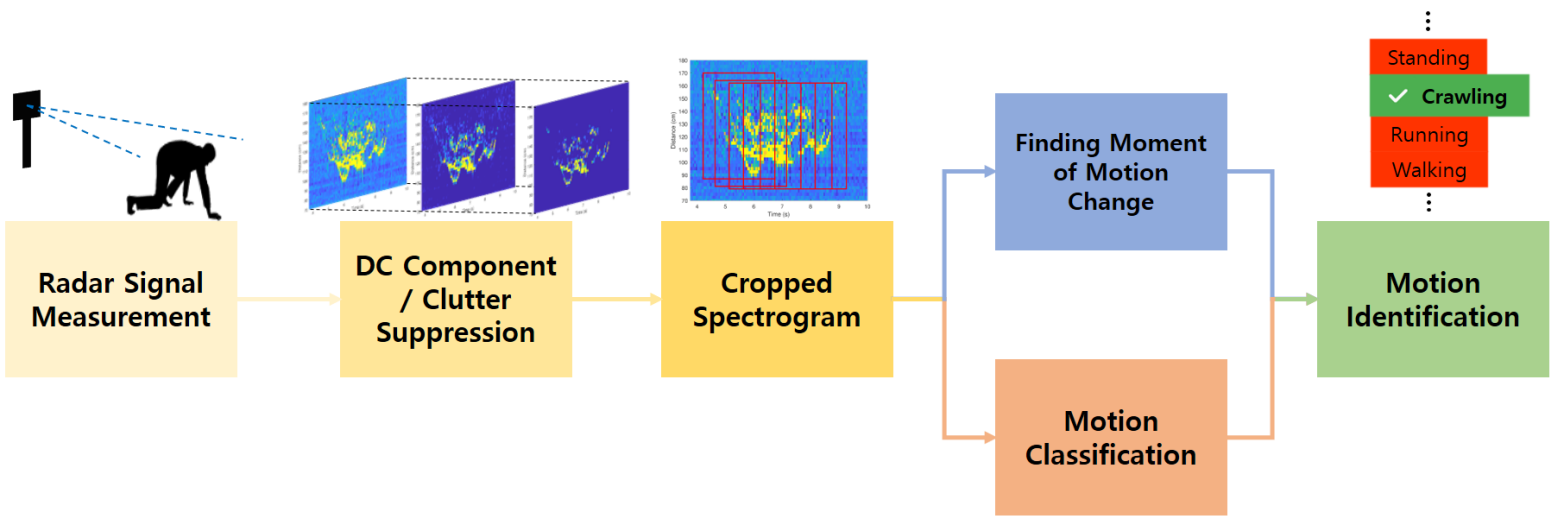
Overall signal processing flow of the proposed method.

The overall signal processing flow of the proposed method is shown in Figure 7. The spectrograms are generated from the received radar signals accumulated over time, and only significant portions are cropped to be used as input data. Those inputs are fed into the networks above and below to determine the moment when the motion changes and to identify the type of motion.

### 3.2. Determining Moment of Motion Change

**Figure 8:**
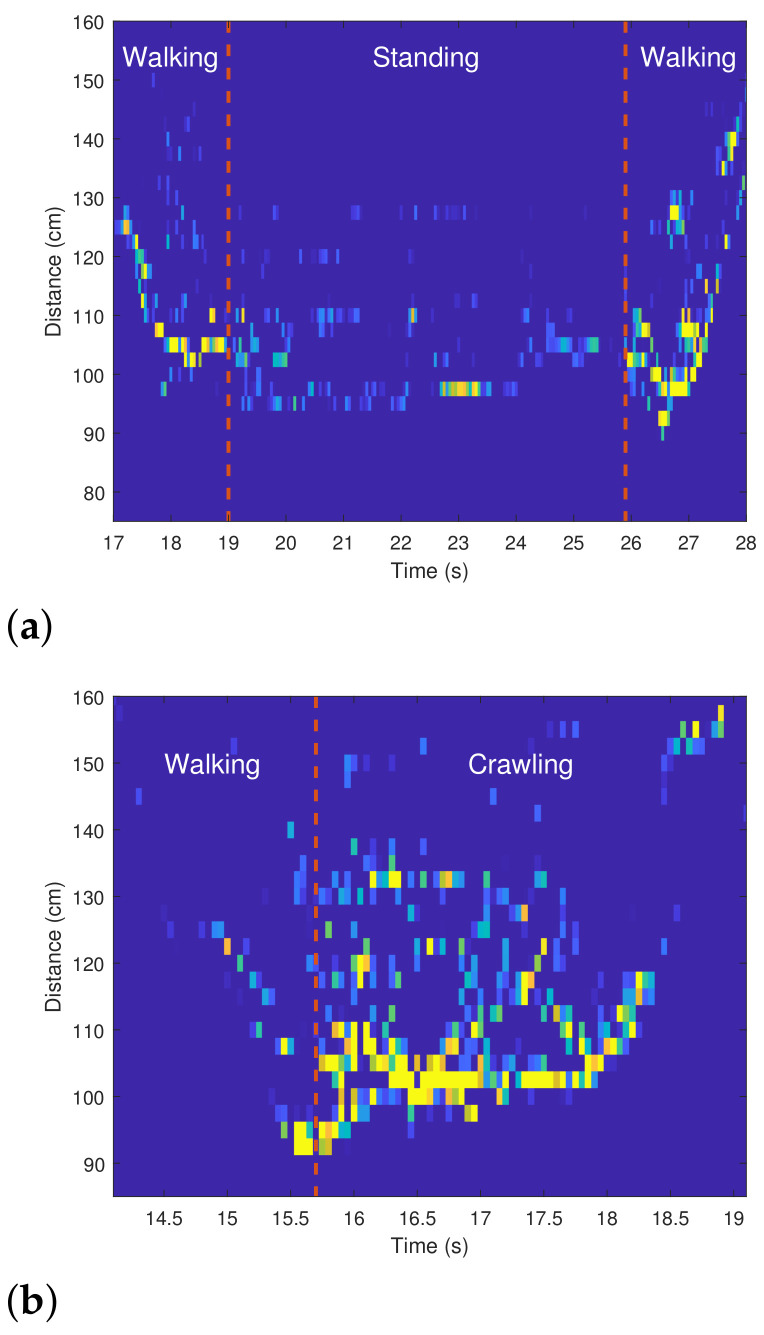
Spectrograms according to changes in human motion: (**a**) From walking to standing, and then walking again. (**b**) From walking to crawling.

Before classifying human motions, we first propose a method of grasping the moment when motion changes. As shown in Figure 8, the shape of the spectrogram changes according to the person’s motion. To find the moment when the motion changes, we can use the statistical characteristics of the signals acquired in each period. For example, the probabilistic moments, such as the mean, variance, skewness, and kurtosis, can be used as measures to characterize the distribution of the detected points in each period [29]. In this study, we used the 1st to 4th order probabilistic moments, which can be calculated as

(6) $$\begin{array}{ccc} M_{1}\left(Z_{c}^{\left(i\right)}\right) & = & \frac{1}{K^{\prime }}\sum_{k^{\prime }=0}^{K^{\prime }-1}Z_{c}^{\left(i\right)}\left[k^{\prime }\right],\\ M_{2}\left(Z_{c}^{\left(i\right)}\right) & = & \frac{1}{K^{\prime }}\sum_{k^{\prime }=0}^{K^{\prime }-1}{\left(Z_{c}^{\left(i\right)}\left[k^{\prime }\right]-M_{1}\left(Z_{c}^{\left(i\right)}\right)\right)}^{2},\\ M_{3}\left(Z_{c}^{\left(i\right)}\right) & = & \frac{\frac{1}{K^{\prime }}\sum_{k^{\prime }=0}^{K^{\prime }-1}{\left(Z_{c}^{\left(i\right)}\left[k^{\prime }\right]-M_{1}\left(Z_{c}^{\left(i\right)}\right)\right)}^{3}}{{\left(M_{2}\left(Z_{c}^{\left(i\right)}\right)\right)}^{\frac{3}{2}}},\\ M_{4}\left(Z_{c}^{\left(i\right)}\right) & = & \frac{\frac{1}{K^{\prime }}\sum_{k^{\prime }=0}^{K^{\prime }-1}{\left(Z_{c}^{\left(i\right)}\left[k^{\prime }\right]-M_{1}\left(Z_{c}^{\left(i\right)}\right)\right)}^{4}}{{\left(M_{2}\left(Z_{c}^{\left(i\right)}\right)\right)}^{2}}, \end{array}$$

where $Z_{c}^{\left(i\right)}\left[k^{\prime }\right]$ is the $k^{\prime }$th element of the cropped signal vector $Z_{c}^{\left(i\right)}$ and $K^{\prime }$ is the length of $Z_{c}^{\left(i\right)}$. As mentioned in Section 3.1, $K^{\prime }$ becomes 40 in our cropped spectrograms.

**Figure 9:**
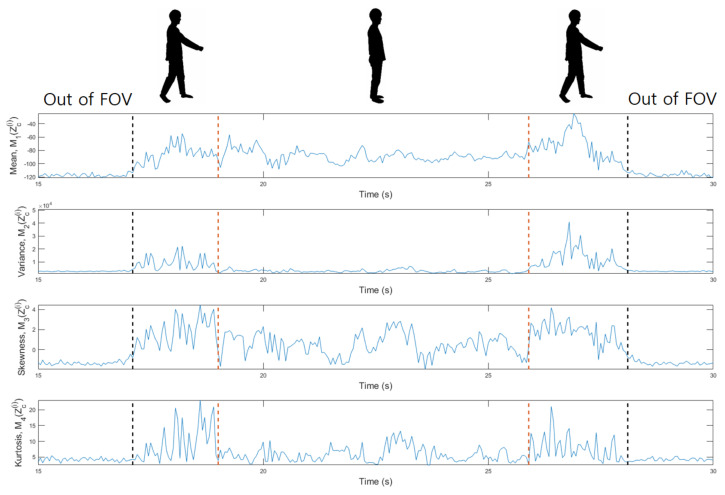
Changes in the values of four probabilistic moments according to the change of motion.

As a result that the number of detected points and the distance to them in each period change with the motions, the moment at which the motion changes can be found by calculating the values of the variables of Equation (6). Figure 9 shows the changes in the values of the four probabilistic moments for the spectrograms of Figure 8a. As shown in Figure 9, when a person enters the FOV of the radar, the values of the four probabilistic moments start to increase. Then, at the boundary between the two motions, the characteristics of the distribution change significantly.

**Figure 10:**
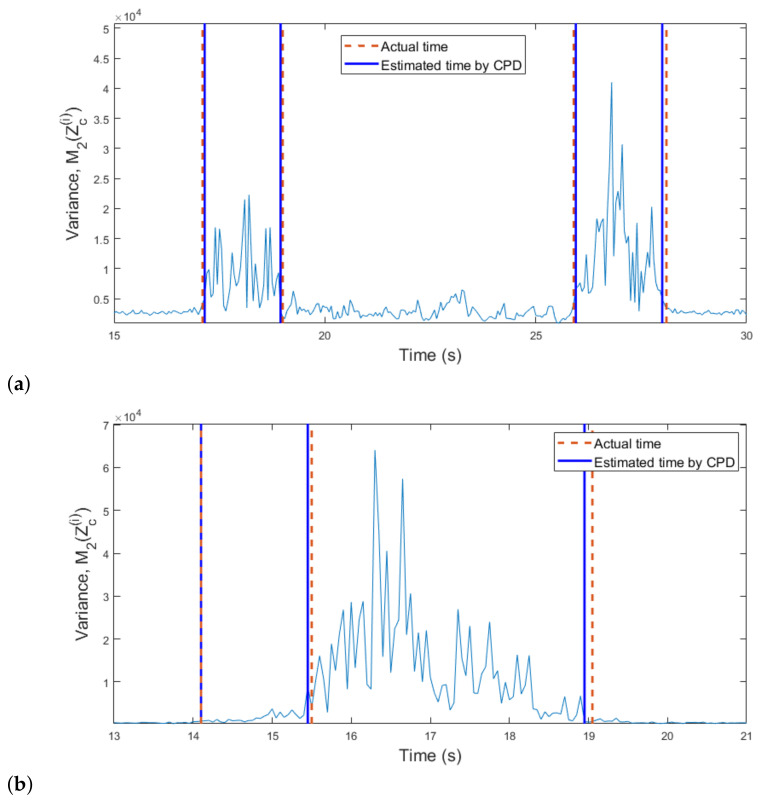
Boundary time between two motions estimated through the change point detection (CPD) algorithm: (**a**) From walking to standing, and then walking again. (**b**) From walking to crawling.

Of the four moments, we confirmed that the 2nd order moment (i.e., variance) has the most pronounced change in its value depending on the motion. Thus, to find the boundary time between two motions, we applied the change point detection (CPD) algorithm [30] to $M_{2}\left(Z_{c}^{\left(i\right)}\right)$. The CPD is a method of finding the point at which the characteristic of the distribution changes in time series data, and can be applied directly to the four moment values we calculated. Figure 10a shows the result of applying the CPD algorithm to the values of $M_{2}\left(Z_{c}^{\left(i\right)}\right)$ in Figure 9. As shown in the figure, the CPD algorithm accurately finds the moments when the motion changes and the absolute time estimation error was 62.5 ms for this case. In addition, we also calculated $M_{2}\left(Z_{c}^{\left(i\right)}\right)$ for the spectrograms in Figure 8b and then applied the CPD algorithm. As shown in Figure 10b, the boundary time between the walking and crawling was found without significant error.

Table 1 shows the mean absolute percentage error for the estimation of the boundary time between two consecutive motions. Looking at Figure 8, the shapes of the spectrograms for walking and standing are obviously different, but those for crawling and walking are quite similar. Thus, as shown in the table, finding the boundary time between walking and crawling is the most difficult. In addition, the spectrograms of walking and running have similarly shaped spectrograms, and they have a distinct difference between the spectrograms of standing. Therefore, the boundary between standing and other motions is relatively clear. In our entire data set, the estimation results for the boundary time exhibited an accuracy of at least 97%.

### 3.3. CNN-Based Motion Classification

In our work, we use a CNN-based classifier to discriminate human motions. In general, because the CNN is trained with images, we used the cropped radar spectrogram described in Section 3.1 as input. In addition, as mentioned in Section 2.2, we accumulated more than 500 spectrograms for each case of performing only one motion or two motions in a row. Then, 70%, 15%, and 15% of entire spectrograms are randomly selected as the training data set, validation data set, and test data set, respectively. First, the CNN structure is trained through the backpropagation process with the training data set. Next, the validation data set is used to mitigate the trained CNN structure being too biased on the training data set. Finally, the test data set is used to evaluate the classification accuracy of the trained CNN structure.

In the classification process, we designed the CNN structures for each of the following three cases and evaluated their performance:

(1) For single motions.
(2) For single motions and successive motions (assigning successive motions to one and the same class).
(3) For single motions and successive motions (assigning successive motions to different classes).

**Figure 11:**
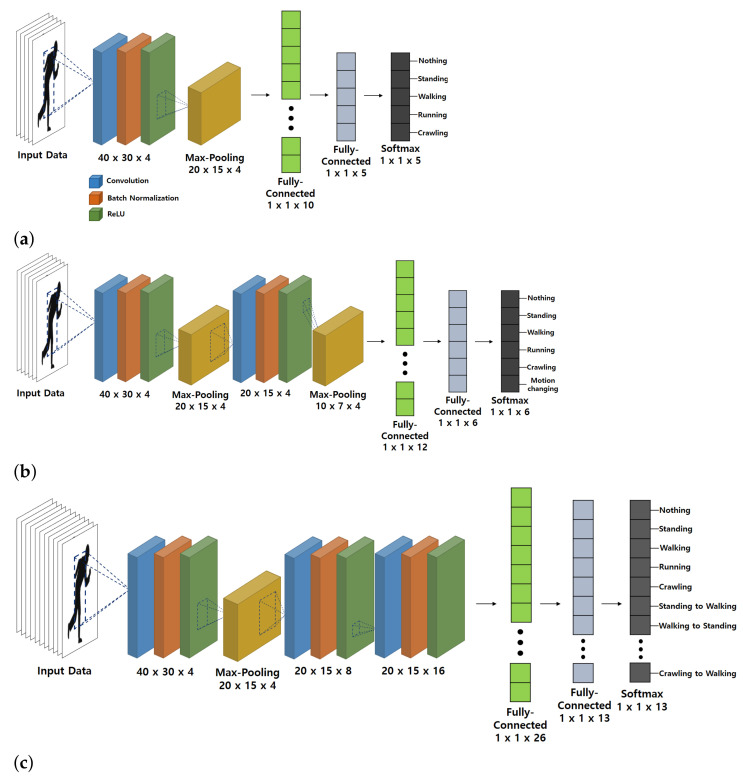
CNN structures used in this study: (**a**) For Case 1. (**b**) For Case 2. (**c**) For Case 3.

The CNN structures designed for each case are depicted in Figure 11. Each structure consists of an input layer, several convolutional layers, pooling layers, fully-connected layers, and an output layer.

First, at the convolutional layer, the input spectrogram is multiplied with the filter. Then, the filter output is passed to the next layer, which can be expressed as

(7) $$\begin{array}{c} O=f_{a}\left(Z_{c}^{\left(N_{p}\right)}*W+b\right), \end{array}$$

where $Z_{c}^{\left(N_{p}\right)}$ is the cropped input spectrogram, W and b are the weight and bias coefficients of the filter. In addition, we used the rectified linear unit (ReLU) function as the activation function, which is represented by $f_{a}$ in Equation (7). Then, a downsampling is conducted in the pooling layer to compress the size of the filter output and avoid overfitting. In our network, we use the maximum pooling, which implies that the output of the filter is divided into several sub-areas, and the maximum values are extracted from each sub-area. The process of convolution, batch normalization, and max pooling is conducted as many times as the number of convolutional layers to obtain features while reducing the size of the spectrogram. The output of the pooling layer is delivered to the fully-connected layer, and the motion classification is performed on that layer. Finally, the softmax layer coverts the calculated values into a form of probability. If we represent the input vector to the softmax layer and the number of classes as $p={\left[q_{1},\;q_{2},\;\ldots ,\;q_{C}\right]}^{T}$ and *C*, the output of this layer can be expressed as

(8) $$\begin{array}{ccc} \hat{p} & = & {\left[\frac{e^{q_{1}}}{\sum_{c=1}^{C}e^{q_{c}}},\;\frac{e^{q_{2}}}{\sum_{c=1}^{C}e^{q_{c}}},\;\ldots ,\;\frac{e^{q_{C}}}{\sum_{c=1}^{C}e^{q_{c}}}\right]}^{T}\\ & = & {\left[{\hat{p}}_{1},\;{\hat{p}}_{2},\;\ldots ,\;{\hat{p}}_{C}\right]}^{T}. \end{array}$$

In addition, we define the cross entropy between the true value $p={\left[p_{1},\;p_{2},\;\ldots ,\;p_{C}\right]}^{T}$ and the estimated value $\hat{p}$ as a loss function, which can be expressed as

(9) $$\begin{array}{c} L=-\sum_{c=1}^{C}{\hat{p}}_{c}\log p_{c}. \end{array}$$

In this study, we assign values to the true value vector p using the one-hot encoding scheme. For example, the classes ‘standing’ and ‘walking’ can be encoded as ${\left[0,\;1,\;0,\;\ldots \right]}^{T}$ and ${\left[0,\;0,\;1,\;\ldots \right]}^{T}$, respectively. Then, while calculating the derivative of L with respect to W for each iteration, the weight update process is performed. This update process can be represented as follows:

(10) $$\begin{array}{c} W^{+}=W-\eta \frac{\partial L}{\partial W}, \end{array}$$

where η denotes the step size in each iteration. This weights update is performed several times until W is properly trained.

In addition, to train the CNN structure suitable in each case, we adopted the batch gradient descent [31]. In this optimization technique, the training is accomplished by splitting the training data set into several batches. The total number of spectrograms used from Cases 1 to 3 are 5111, 13,176, and 13,176, respectively, and the number of classes to be distinguished varies from case to case. Thus, from Cases 1 to 3, different batch sizes of 150, 300, and 100 were used. In addition, the number of iterations became 920, 1200, and 3680 times for each case. In addition, after the training data set was trained 40 times, the network training has ended. In this process, the step size η, which is also known as the learning rate, was set to 0.0001.

**Figure 12:**
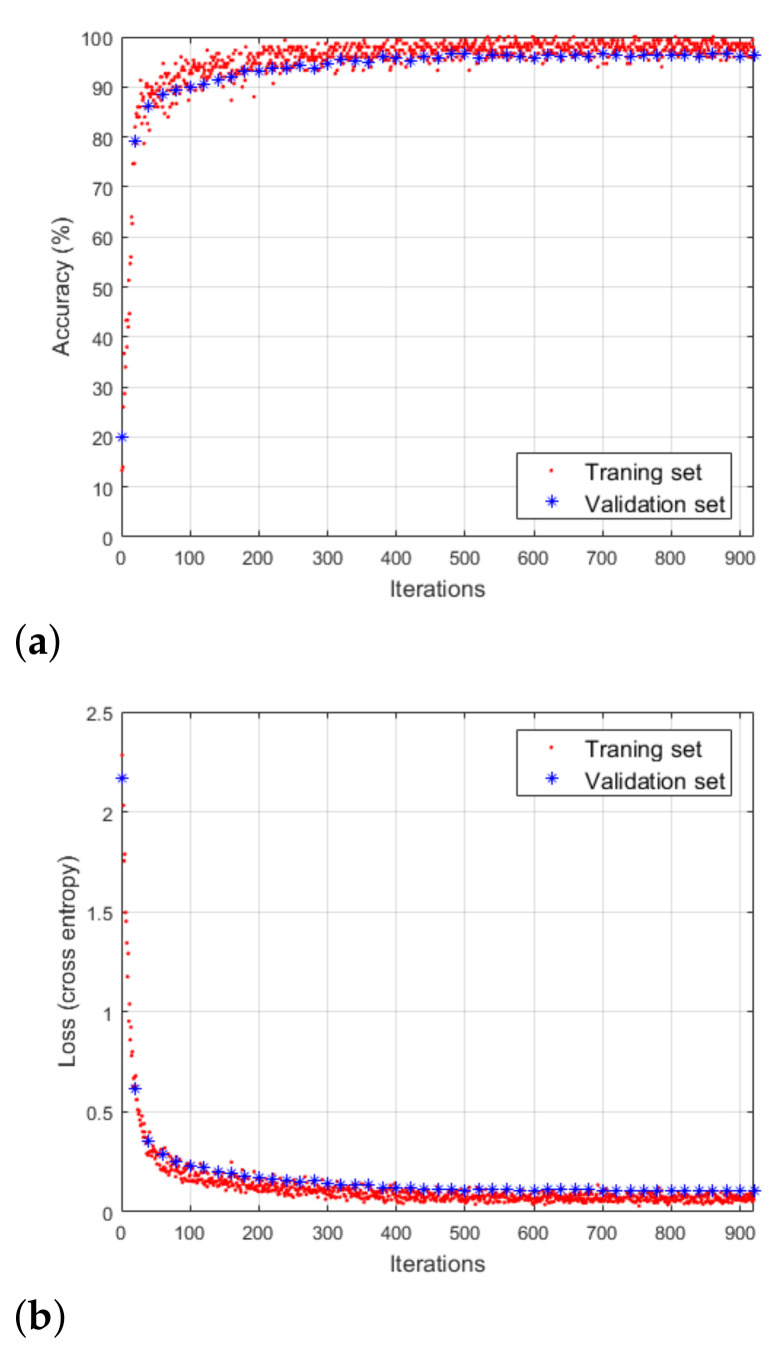
Performance evaluation of the CNN-based classifier used in Case 1: (**a**) Classification accuracy. (**b**) Loss value.

For Case 1, the classification performance and the loss value with respect to the number of iterations are shown in Figure 12. To validate the performance of the CNN-based classifier, the network validation was conducted every 20 iterations. As shown in the figure, as the loss in training decreases, the classification accuracy conversely increases. After performing 250 iterations, the accuracy and the loss each converge to a specific value and there was no further enhancement in the performance of the CNN-based classifier. In other words, through sufficient learning process, the loss value reached the local minimum value and the derivative value of the loss became almost zero. At the point of convergence, the classification accuracy derived from the training data set and the validation data set was 99.33% and 97.39%, and the loss values from them were 0.034 and 0.087, respectively. Moreover, the classification accuracy and the loss value exhibited similar trends in the training data set and the validation data set. It means that the trained CNN structure is not biased towards the training data set. Similarly for Cases 2 and 3, the evaluation and analysis of classification performance can be done equally. Figure 11 shows the CNN structures derived through this process for three cases. After several performance evaluations by adjusting the hyper-parameters of the network, the three simplest structures with a classification performance over 95% were determined. In general, as the number of classes to be classified increases or the form of the input data becomes more complex, the CNN structure tends to become more complex. In that context, when the number of motions to be classified increases, the structure of the CNN becomes increasingly complex, as shown in Figure 11. In addition, unlike general RGB images, spectrograms are 1-dimensional matrix data. Thus, even a relatively simple CNN structure can show high target classification performance for radar data sets.

**Figure 13:**
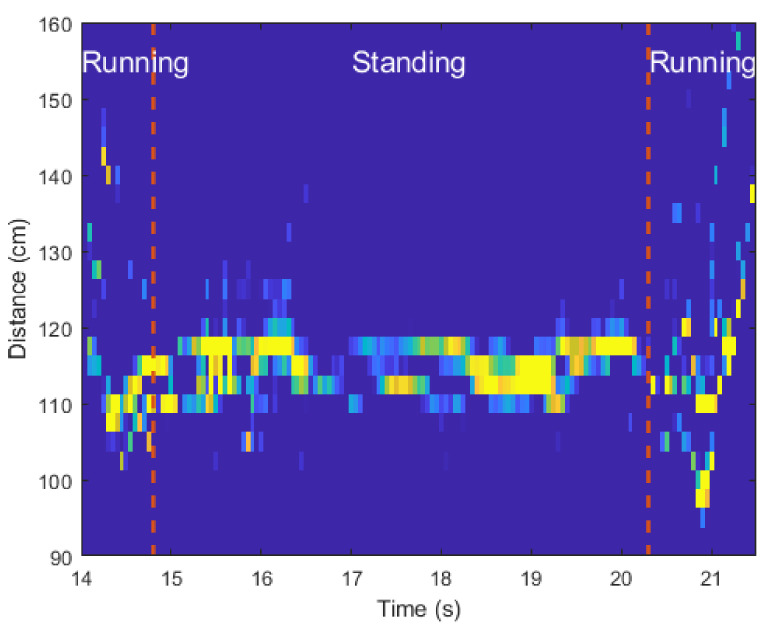
Spectrogram according to changes in human motion: From running to standing, and then running again.

In addition, Table 2, Table 3 and Table 4 show the confusion matrices for the classification results of each CNN-based classifier. First, looking at the classification results in Table 2, the network we designed confuses walking and crawling the most. This is because the spectrograms in these two cases are similar, compared to other motions. For Case 2, all spectrograms corresponding to the motion change were grouped into one class, and motion classification was performed. As shown in Table 3, the spectrograms for the part where the movement changes were classified with high accuracy because they have completely different shapes than spectrograms of a single motion. Finally, Table 4 shows the classification accuracy of the CNN structure after dividing the spectrograms for successive motions into subdivided classes. As shown in Table 4, single actions were classified with high accuracy, and most of the classification errors occurred when classifying the spectrograms for performing two consecutive motions. Looking at the classification results in Table 4, the classification accuracy for M7 and M9 is the lowest. This is because the spectrograms between walking and standing have almost similar shapes to the spectrograms between running and standing, as can be seen in Figure 8a and Figure 13. When compared with the results in Table 3, the classification accuracy was lower than when spectrograms for continuous motions were designated as one class. However, because the number of convolutional layers increased, the classification accuracy was still over 95% on average.

## 4. Conclusions

In this paper, we proposed a method of identifying the moment when a person’s motion changes and discriminating the type of motion using radar sensor data. In our experiment, we used a small radar sensor in the millimeter wave band with high range resolution. In addition, we used the cropped radar spectrogram to understand the characteristics of human motion over time. First, to find the moment when the motion changes, we used the probabilistic moments, such as the mean, variance, skewness, and kurtosis, representing statistical characteristics of received radar signals. In particular, when the CPD was applied to the variance, the moment when the motion was changed was found with an error rate of within 3% on average. Then, a CNN-based classifier was used to determine the type of motion. In this process, we designed three different CNN structures suitable for the type of input. We evaluated the performance of the CNN structures for spectrograms of single motions and continuous motions, and the classification accuracy was more than 95%. The proposed method is expected to be used with camera sensors to detect and monitor the movement of people indoors.

## Figures and Tables

**Table 1:** Mean absolute percentage error for the estimation of the boundary time.

Consecutive Motions	Mean Absolute Percentage Error
Standing and running	1.5 %
Standing and walking	1.25 %
Walking and running	2.25 %
Walking and crawling	3.75 %

**Table 2:** Classification results from the CNN-based classifier (Case 1).

Actual Motion	Predicted Motion				
	Nothing	Standing	Walking	Running	Crawling
Nothing	100%	0%	0%	0%	0%
Standing	0%	100%	0%	0%	0%
Walking	0%	0%	90.85%	1.44%	11.83%
Running	0%	0%	0%	98.56%	0%
Crawling	0%	0%	9.15%	0%	88.17%

**Table 3:** Classification results from the CNN-based classifier (Case 2).

Actual Motion	Predicted Motion					
	Nothing	Standing	Walking	Running	Crawling	Motion Changing
Nothing	100%	0%	0%	0%	0%	0%
Standing	0%	98%	1.2%	0%	0%	0.16%
Walking	0%	0%	84.94%	0.78%	7.48%	0.16%
Running	0%	0%	1.2%	99.22%	0%	0.08%
Crawling	0%	1.33%	9.64%	0%	92.52%	0.08%
Motion changing	0%	0.67%	3.01%	0%	0%	99.51%

**Table 4:** Classification results from the CNN-based classifier (Case 3).

Actual Motion	Predicted Motion												
	(M1)	(M2)	(M3)	(M4)	(M5)	(M6)	(M7)	(M8)	(M9)	(M10)	(M11)	(M12)	(M13)
Nothing (M1)	100%	0%	0%	0%	0%	0%	0%	0%	0%	0%	0%	0%	0%
Standing (M2)	0%	100%	0%	0%	0%	0%	0%	0%	0%	0%	0%	0%	0%
Walking (M3)	0%	0%	100%	0.61%	0.72%	0%	0%	0%	0%	0%	0%	0%	0%
Running (M4)	0%	0%	0%	99.39%	0%	0%	0%	0%	0%	0%	0%	0%	0%
Crawling (M5)	0%	0%	0%	0%	99.28%	0%	0%	0%	0%	0%	0%	0%	0%
Standing to walking (M6)	0%	0%	0%	0%	0%	98.08%	0%	1.3%	0%	0%	0%	0%	0%
Walking to standing (M7)	0%	0%	0%	0%	0%	0%	94.24%	0%	8.2%	0%	0%	0%	0%
Standing to running (M8)	0%	0%	0%	0%	0%	1.92%	0%	98.05%	0%	0%	0%	0%	0%
Running to standing (M9)	0%	0%	0%	0%	0%	0%	5.76%	0%	90.71%	0%	0%	0%	0%
Walking to running (M10)	0%	0%	0%	0%	0%	0%	0%	0.65%	0%	95.42%	0%	1.85%	0.7%
Running to walking (M11)	0%	0%	0%	0%	0%	0%	0%	0%	1.09%	3.27%	98.56%	1.85%	1.41%
Walking to crawling (M12)	0%	0%	0%	0%	0%	0%	0%	0%	0%	1.31%	0%	94.44%	0%
Crawling to walking (M13)	0%	0%	0%	0%	0%	0%	0%	0%	0%	0%	1.44%	1.85%	97.89%

## Data Availability

Data sharing not applicable.

## Abbreviations

The following abbreviations are used in this manuscript:

ADC	Analog-to-digital converter
CNN	Convolutional neural network
CPD	Change point detection
DC	Direct current
DSP	Digital signal processor
FM	Frequency mixer
FMCW	Frequency-modulated continuous wave
FOV	Field of view
LPF	Low-pass filter
ReLU	Rectified Linear Unit
RGB	Red, green, and blue
Rx	Receiving antenna
Tx	Transmit antenna
UWB	Ultra-wideband
VCO	Voltage-controlled oscillator
WG	Waveform generator
